# Nasal Delivery of an Adenovirus-Based Vaccine Bypasses Pre-Existing Immunity to the Vaccine Carrier and Improves the Immune Response in Mice

**DOI:** 10.1371/journal.pone.0003548

**Published:** 2008-10-29

**Authors:** Maria A. Croyle, Ami Patel, Kaylie N. Tran, Michael Gray, Yi Zhang, James E. Strong, Heinz Feldmann, Gary P. Kobinger

**Affiliations:** 1 Division of Pharmaceutics, College of Pharmacy, The University of Texas at Austin, Austin, Texas, United States of America; 2 Institute of Cellular and Molecular Biology, The University of Texas at Austin, Austin, Texas, United States of America; 3 Special Pathogens Program, National Microbiology Laboratory, Public Health Agency of Canada, Winnipeg, Canada; 4 Department of Medical Microbiology, University of Manitoba, Winnipeg, Canada; 5 Department of Internal Medicine, Division of Immunology, University of Michigan, Ann Arbor, Michigan, United States of America; 6 Department of Pediatrics and Child Health, University of Manitoba, Winnipeg, Canada; Queensland Institute of Medical Research, Australia

## Abstract

Pre-existing immunity to human adenovirus serotype 5 (Ad5) is common in the general population. Bypassing pre-existing immunity could maximize Ad5 vaccine efficacy. Vaccination by the intramuscular (I.M.), nasal (I.N.) or oral (P.O.) route with Ad5 expressing Ebola Zaire glycoprotein (Ad5-ZGP) fully protected naïve mice against lethal challenge with Ebola. In the presence of pre-existing immunity, only mice vaccinated I.N. survived. The frequency of IFN-γ+ CD8+ T cells was reduced by 80% and by 15% in animals vaccinated by the I.M. and P.O. routes respectively. Neutralizing antibodies could not be detected in serum from either treatment group. Pre-existing immunity did not compromise the frequency of IFN-γ+ CD8+ T cells (3.9±1% naïve vs. 3.6±1% pre-existing immunity, PEI) nor anti-Ebola neutralizing antibody (NAB, 40±10 reciprocal dilution, both groups). The number of INF-γ+ CD8+ cells detected in bronchioalveolar lavage fluid (BAL) after I.N. immunization was not compromised by pre-existing immunity to Ad5 (146±14, naïve vs. 120±16 SFC/million MNCs, PEI). However, pre-existing immunity reduced NAB levels in BAL by ∼25% in this group. To improve the immune response after oral vaccination, the Ad5-based vaccine was PEGylated. Mice given the modified vaccine did not survive challenge and had reduced levels of IFN-γ+ CD8+ T cells 10 days after administration (0.3±0.3% PEG vs. 1.7±0.5% unmodified). PEGylation did increase NAB levels 2-fold. These results provide some insight about the degree of T and B cell mediated immunity necessary for protection against Ebola virus and suggest that modification of the virus capsid can influence the type of immune response elicited by an Ad5-based vaccine.

## Introduction

The ability of human adenoviruses to induce strong innate and adaptive immune responses makes them powerful adjuvants that facilitate the immune response against an encoded antigen. Recombinant adenoviruses have been shown to elicit significant immune responses to bacterial (anthrax, plague), viral (Hepatitis C, Rabies, SARS) and tumour-associated antigens [1]–[3]. While these results are encouraging, immunity eventually develops against virus capsid proteins. This severely reduces the immunogenicity of adenovirus-based vaccines in mice, [4]–[8], primates [9] and humans [10]. This problem is also significant since a large portion of the Western world has marked levels of anti-adenovirus serotype 5 (Ad5) antibodies and is also prominent in regions of sub-Saharan Africa and Southeast Asia, where many of these vaccines are needed [11], [12]. Thus, assessment of the impact of pre-existing immunity on immune protection and alternative vaccination strategies may be needed for successful use of many adenovirus-based vaccines.

Several strategies have been developed to address the prevalence of pre-existing immunity to Ad5 in the general population. Increasing the vector dose or adopting a prime-boost regimen in order to overcome pre-existing immunity to the virus is a common approach [2]. There is mixed enthusiasm for this plan, however, due to the documented toxicity associated with high doses of adenovirus and the length of time required for prime-boost regimens when only a prime could be sufficient [13], [14]. ‘Seroswitching’, using recombinant adenoviruses constructed from chimpanzees or rare human serotypes with limited exposure rates such as adenoviruses 35 and 11 can elicit potent immune responses that are minimally affected by pre-existing immunity [7], [8], [15]–[21]. Hexon-chimeric adenoviruses can also avoid neutralization [22], [23]. Both approaches offer promise in the context of addressing pre-existing immunity, but require further investigation in response to concerns regarding safety and feasibility of large-scale production. Covalent attachment of polyethylene glycol or incorporation of the virus into polymer matricies can also effectively protect Ad5 from neutralization [24]–[31]. Delivery of Ad5-based vaccines by mucosal routes can also circumvent the effect of pre-existing immunity and induce a significant immune response against an encoded antigen [32].

Recombinant adenoviruses are one of the few well-studied vectors currently under development for vaccination against Ebola virus infection. The first protocol for an Ebola vaccine employed a prime-boost regimen consisting of naked DNA expressing either Ebola glycoprotein (GP) or nucleoprotein (NP) and recombinant Ad5 expressing Ebola GP to successfully protect non-human primates against a lethal challenge of Ebola [33]. This has since led to several Phase I clinical trials [34], [35] in which each component of the vaccine is administered by intramuscular injection. To date, there have been only two reports describing mucosal administration of an Ebola vaccine [36], [37]. Nasal administration of recombinant human parainfluenza virus type 3 (HPIV3) vectors expressing Ebola GP and/or NP to Guinea pigs and rhesus monkeys conferred complete protection against a lethal challenge with Ebola.

We have previously found that a single dose of a recombinant adenovirus expressing Ebola Zaire GP given by either the oral or the nasal route is capable of affording protection against lethal challenge in naïve mice and that mucosal immunization can stimulate a broad, prolonged T cell-mediated immune response in both the systemic and mucosal compartments [37]. The primary objective of this study was to test the hypothesis that administration of an Ad5-based Ebola vaccine by either the nasal or oral route can circumvent pre-existing immunity and confer full protection upon challenge. Systemic and mucosal T and B cell responses to Ebola GP were assessed in both naïve mice and those with pre-existing immunity. The influence of PEGylation of the vaccine carrier on the immune response after oral immunization is also described.

## Materials and Methods

### Construction, production and formulation of adenoviral vectors

The E1/E3-deleted adenovirus vector expressing the Ebola Zaire glycoprotein was created by cloning the open reading frame (ORF) sequence of the glycoprotein in the plasmid pShuttle (Adeno-X Expression system I, BD Clonetech, Palo Alto, CA) for subsequent insertion in the E1 region of the human adenovirus serotype 5 genome. The human cytomegalovirus (CMV) promoter included in the Adeno-X expression system was used to drive the expression of the Ebola Zaire glycoprotein in the final recombinant adenovirus serotype 5 construct. Authenticity of the final product was confirmed by sequencing of the recombinant virus rescued by transfecting the linearized DNA into 293 cells. Virus was sequentially amplified to large-scale infections (5×10^8^ cells) and purified on an affinity column (Adeno-X virus purification mega kit, BD Clonetech, Mountain View, CA) according to the manufacturer's instructions. Genome structures of vectors were analyzed by restriction digestion of isolated viral DNA and compared with those of the original molecular clones. Particle number and infectivity of vectors were determined by standard optical density reading and immunodetection of the hexon protein, respectively, following infection of 293 cells with limiting dilutions of each vector preparation according to the recommendations by the manufacturer (Adeno-X rapid titer kit, Clontech, Mountain View, CA). Purified virus was administered in sterile phosphate buffered saline (pH 7.4) and had particle to plaque forming unit (pfu) ratios of 100∶1 or less.

### PEGylation of recombinant adenovirus

First generation recombinant adenovirus expressing Ebola Zaire glycoprotein was prepared and purified as described above. The protein content of the virus preparation was determined using BioRad DC Protein Assay reagents (BioRad, Hercules, CA) and bovine serum albumin as a standard in a microplate format. According to established protocols, 10 µg of monomethoxypoly(ethylene) glycol, activated by tresyl chloride (Sigma Aldrich, St. Louis, MO), was added for each microgram of protein present [38]. The coupling reaction was performed at 25°C with gentle agitation. The reaction was stopped by the addition of L-lysine, in a 10-fold excess with respect to the amount of PEG added. Unreacted PEG, excess L-lysine, and reaction byproducts were removed by buffer exchange over a second Econo-Pac 10DG disposable chromatography column equilibrated with 100 mM potassium phosphate-buffered saline (pH 7.4). PEGylated preparations were administered in sterile potassium phosphate buffered saline (pH 7.4) and had particle to plaque forming unit (pfu) ratios of 100∶1 or less as determined by the Adeno-X rapid titer kit. Characterization of these preparations revealed significant changes in biophysical properties of the virus once the reaction was complete such as the PEG-Dextran partition coefficient and peak elution times during capillary electrophoresis (as described previously [30], [38]). Approximately 13,000 PEG molecules were associated with each virus particle in the studies outlined here as determined by a PEG-biotin assay [30].

### Immunization of mice and challenge

B10.BR mice were immunized with 1×10^10^ particles of recombinant virus per mouse either by intramuscular injection (50 µl) in the right hindlimb, or by oral gavage (100 µl) using oral feeding needles (18G, 2.25 mm dia., Popper & Sons, Inc, New Hyde Park, NY). For nasal immunization, mice were anesthetized with isoflurane. Once anesthesia was achieved, 1×10^10^ particles of virus slowly delivered as a bolus into the nostrils using a standard micropipette (Gilson, Middleton, WI) as previously described [39].

Pre-existing immunity to adenovirus serotype 5 was established by injecting 5×10^10^ particles of adenovirus expressing beta-galactosidase (AdlacZ) by intramuscular injection in the right hindlimb 30 days prior to vaccination with Ad5-ZGP. This protocol has been documented to activate T and B cells against virus capsid proteins and elicit humoral immunity [8], [11]. At the time of vaccination, mice had an average anti-adenovirus circulating NAB titer of 1∶320, which falls within lower range of average values reported in humans after natural infection [11]. Mice were challenged by intraperitoneal injection of 200× LD_50_ of mouse-adapted Ebola virus, Zaire strain (MA-ZEBOV) in 200 µl sterile saline [40]. After challenge, the animals were weighed daily for 13 days and monitored for clinical signs of Ebola infection using an approved scoring sheet. All procedures and the scoring method were approved by the Institutional Animal Care Committee at the National Microbiology Laboratory (NML) of the Public Health Agency of Canada (PHAC) according to the guidelines of the Canadian Council on Animal Care. All infectious work was performed in the ‘Biosafety Level 4’ (BSL4) facility at NML, PHAC.

### Antibody detection assays

#### A) Anti-ebola neutralizing antibody (serum)

Sera collected from immunized mice were inactivated at 56°C for 45 minutes. Serial dilutions of each sample (1∶10, 1∶20, 1∶40, etc, in 50 µl of DMEM) were mixed with equal volumes of recombinant Ebola Zaire expressing the enhanced green fluorescent protein (EGFP) reporter gene (ZEBOV-EGFP, 100 transducing units/well, according to EGFP expression) and incubated at 37°C for 90 minutes [41]. The mixture was then added to subconfluent VeroE6 cells in 96-well flat-bottomed plates and incubated for 5–10 minutes at room temperature. Control wells were infected with equal amounts of either ZEBO-EGFP with media without serum or that containing non-immune serum. 100 µl of DMEM supplemented with 20% FBS was then added to each well and plates were incubated at 37°C in 5% CO_2_ for 48 hr. Cells were subsequently fixed with 10% buffered formalin for 24 h and examined under a fluorescent microscope. Sample dilutions which showed >50% reduction in the number of green cells compared to controls scored positive for neutralizing antibody.

#### B) Anti-ebola neutralizing antibody (mucosal)

For the evaluation of the specific levels of IgG and IgA antibodies, bronchoalveolar lavage (BAL) fluid was collected *in situ* with a 20-gauge catheter inserted into the proximal trachea, flushing the lower airways three times with 1 milliliter of L15 media (Sigma). BAL from each animal was incubated at 56°C for 45 minutes. Two-fold serial dilutions were added to 96 well plates pre-coated overnight with 30 ng of ZEBOV-like particles per well and incubated at 37°C for 1 hr. Goat anti-mouse secondary antibody conjugated to horseradish peroxidase (HRP) was then added and the plate was incubated for one additional hour at 37°C. The ABTS Peroxidase Substrate System (KPL) was used for detection and data collected using an ASYS UVM 340 ELISA plate reader (Isogen Life Science) at OD405. All infectious *in vitro* work was performed in the BSL4 laboratory at the NML, PHAC.

#### C) Anti-adenovirus serotype 5 neutralizing antibody (serum)

Pre-existing immunity against recombinant adenovirus 5 was assessed by determining the amount of neutralizing antibody present in serum according to established methods [30]. In brief, serum was incubated at 56°C for 30 minutes and then diluted in DMEM in twofold increments starting from a 1∶20 dilution. Each dilution (100 µl) was mixed with an aliquot of a standard stock of adenovirus type 5 expressing *E. coli* beta-galactosidase (10^6^ pfu), incubated for 1 hour at 37°C, and applied to HeLa cells in 96-well plates (2×10^4^ cells/well). One hundred microliters of DMEM supplemented with 20% FBS was then added to each well. Cells were incubated at 37°C for 24 hours. Neutralizing antibody titers were calculated as the highest dilution at which 50% of the cells stained blue by visual inspection.

### Frequency of INF-γ positive cells

For the evaluation of INF-γ positive CD8+ T cells 10 days post-immunization, splenocytes were harvested and cultured (1×10^6^/sample) for 5 hours at 37°C in 96-well round bottom microtiter plates in DMEM supplemented with 10% FBS, 2-beta-mercaptoethanol (10^−6^ M) and GolgiStop (1 µl/ml, BD PharMingen, San Diego, CA). The TELRTFSI peptide which carries the Ebola Zaire GP immunodominant MHC class I epitope for mice of the H-2^k^ haplotype (B10.BR) was used for stimulation at a concentration of 1 µg/ml [42]. Control cells were treated with either an unrelated peptide or no peptide. After washing, cells were stained with 100 µl of a FITC-anti mouse CD8a antibody (1∶100 dilution, PharMingen) at 4°C for 30 minutes. Cells were washed again, permeabilized in 1× Cytofix/Cytoperm (PharMingen) for 20 minutes at 4°C, washed with 1× Perm/Wash (PharMingen) and stained with 100 µl of a PE-anti mouse IFN-γ antibody (1∶100 dilution, PharMingen) in the same buffer at 4°C for 30 minutes.

Quantitation of INF-γ positive CD8+ T cells isolated from splenocytes or mononuclear cells from bronchioalveolar lavage (BAL), mesenteric lymph nodes (MLN) and Peyer's patches (PP) 45 days after vaccination was performed using an ELISPOT assay (ELISPOT Mouse Set, BD PharMingen, San Diego, CA) according to the manufacturer's instructions. Briefly, a 96-well ELISPOT plate was coated with 5 µg/ml anti-mouse IFN-γ capture antibody. Cells pooled from 4 B10.BR mice per experimental group were added to microwells along with the TELRTFSI peptide (2 µg/well). Control cells were incubated either without peptide or with the non-specific stimulator, SEB (200 ng/ml). After incubation with a biotinylated anti-mouse IFN-γ detection antibody and Streptavidin-horseradish peroxidase antibody, wells were counted using an ELISPOT reader (AID EliSpot reader system, Cell Technology, Colombia, MD).

### Statistical analysis

Data were analyzed for statistical significance by performing unpaired T tests (two-tailed p value) or one-way analysis of variance (ANOVA) when appropriate. The differences in the mean or raw values among treatment groups were considered significant when *p*<0.05.

## Results

### Effect of Mucosal Immunization on T and B Cell Responses Against Ebola Glycoprotein in Mice with Pre-Existing Immunity Against Adenovirus

**Figure 1:**
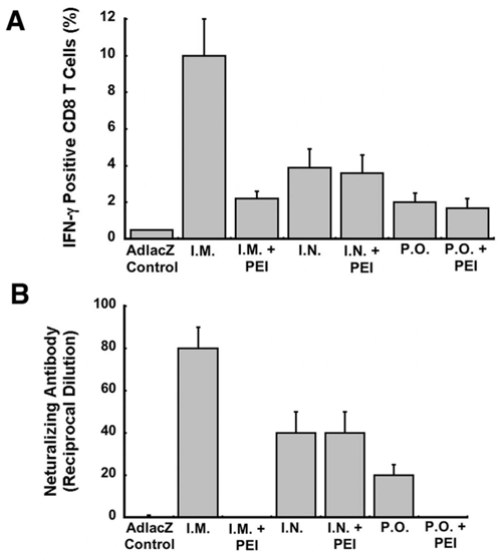
Pre-Existing Immunity Against Adenovirus Does not Compromise the Strength of the Cellular and Humoral Immune Response Against Ebola Glycoprotein After Intranasal Immunization. Naïve mice (n = 10) were vaccinated with a single dose of 1×10^10^ particles of adenovirus expressing the Ebola Zaire glycoprotein (Ad5-ZGP) by the intramuscular (I.M.), nasal (I.N.) or oral (P.O.) route. Animals in which pre-existing immunity (PEI) was established by I.M. injection of 5×10^10^ particles of adenovirus serotype 5 expressing beta-galactosidase (AdlacZ) 30 days prior to vaccination were also immunized in the same manner. At the time of vaccination, mice had an average anti-adenovirus circulating NAB titer of 1∶320. Animals given a single dose of (AdlacZ) served as negative controls (AdlacZ Control). (A) Frequency analysis of IFN-γ secreting CD8+ T cells harvested from splenocytes 10 days post-immunization (n = 4/group). The TELRTFSI peptide, specific for the Ebola Zaire glycoprotein (0.4 µg/well), was incubated with 1×10^6^ splenocytes and cells analyzed by flow cytometry. (B) Neutralizing antibody (NAB) levels against ZEBOV-EGFP were evaluated 25 days post-vaccination (n = 10/group). In both panels, error bars represent the standard deviation of the data.

In an effort to correlate markers of immunity with protection against Ebola infection after mucosal immunization, T and B cell specific immune responses against Ebola glycoprotein were analyzed in mice in the presence or absence of pre-existing immunity (PEI) to adenovirus 10 days after vaccination with a first generation adenovirus serotype 5 vector expressing the Zaire Ebola glycoprotein (Ad5-ZGP). Intracellular staining and flow cytometry (FACS) revealed that Ebola glycoprotein peptide-specific activation of CD8+ T cells, as measured by production of IFN-γ, occurred in naïve animals immunized by the nasal route at a frequency of 3.9±1% (I.N., Figure 1A). Pre-existing immunity was induced by intramuscular administration of recombinant Ad5 expressing a non-relevant antigen, beta-galactosidase (AdlacZ), 30 days prior to vaccination with Ad5-ZGP. At the time of vaccination, mice had an average anti-adenovirus circulating NAB titer of 1∶320. Pre-existing immunity did not significantly alter activation of CD8+ T cells when the vaccine was given intranasally (I.N.+PEI, 3.6±1%, *p* = 0.07). Oral immunization lowered the response against Ebola glycoprotein (P.O., 2±0.5%). Samples obtained from animals with pre-existing immunity and vaccinated in the same manner were barely above the positive threshold (three times the average background level obtained from animals treated with the irrelevant virus, AdlacZ). Samples obtained from naïve animals immunized by the intramuscular route (I.M.) contained the largest population of IFN-γ positive CD8+ T cells (10±2%). Animals treated with the AdlacZ vector alone (AdlacZ-control) served as negative controls and produced 0.5% CD8+, IFN-γ+ T cells in response to the Ebola glycoprotein-specific peptide.

The B cell response against Ebola glycoprotein achieved by administration of the vaccine was determined by incubating a recombinant Ebola virus (Zaire strain) expressing green fluorescent protein (ZEBOV-EGFP) with serum collected 25 days after vaccination [8], [37]. Neutralizing antibody (NAB) levels equivalent to 40±10 reciprocal dilution were detected in both naïve animals and those with pre-existing immunity after intranasal immunization (Figure 1B). Samples from naïve animals immunized by the oral route contained NAB levels equivalent to 20±5 reciprocal dilution whereas anti-Ebola NAB could not be detected in samples obtained from animals immunized by the same route with pre-existing immunity (P.O.+PEI). NAB levels of 80±10 were detected in samples obtained from naïve mice immunized by the intramuscular route. Anti-Ebola NAB could not be detected in samples from mice given the AdlacZ vector alone (AdlacZ Control).

### Effect of Mucosal Immunization on Systemic and Localized T-Cell Mediated Immune Responses Against Ebola Glycoprotein in Mice with Pre-Existing Immunity

**Figure 2:**
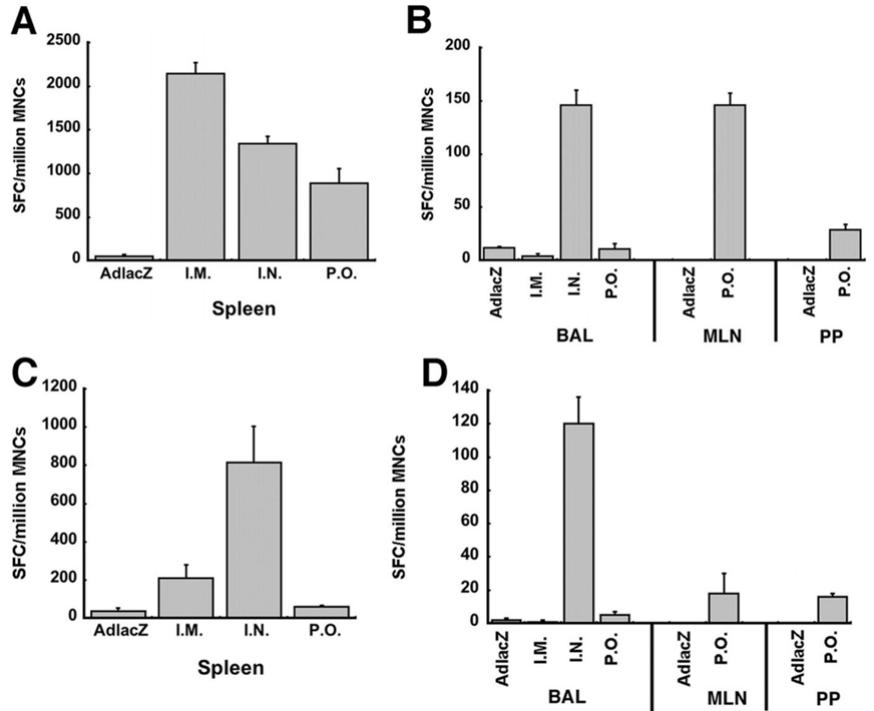
Pre-Existing Immunity Against Adenovirus Does Not Compromise the Systemic or Mucosal Cellular Response Generated Against Ebola Glycoprotein After Intranasal Immunization. The frequency of IFN-γ positive mononuclear cells was analyzed from the spleen (panels A and C), the lung via bronchioalveolar lavage (BAL) and the intestine via the mesenteric lymph nodes (MLN) and Peyer's patches (PP) (panels B and D) 45 days post-immunization by ELISPOT. Samples were obtained from naïve animals (Panels A and B) and those with pre-existing immunity to adenovirus serotype 5 (Panels C and D). Cells were plated at 1×10^5^ or 1×10^4^ cells per well, stimulated with the TELRTFSI peptide and expression of IFN-γ detected with an anti-mouse IFN-γ antibody. Cells isolated from BAL, MLN or PP of four mice were pooled and samples tested in duplicate. In each panel, the number of spot-forming cells (SFC) per million mononuclear cells (MNCs) is shown on the y-axis. Please note - the scale of this axis differs between panels to accent the differences between treatment groups. Error bars represent the standard deviation of the data. Animals immunized with an irrelevant adenoviral vector (AdlacZ) served as negative controls. Positive results obtained from this group are indicative of artificial cellular stimulation that may have occurred during processing and culturing of samples.

Since the mucosa is often the primary sight of exposure, long-term, localized immune responses against Ebola virus are desirable for providing optimal protection against infection. In this context, T cell mediated immune responses were assessed by ELISPOT from various tissue compartments specific to the route of immunization 45 days after vaccination. The number of activated IFN-γ secreting mononuclear cells harvested from splenocytes was significantly reduced by 38 and 59% in mice vaccinated nasally or orally when compared to those immunized by intramuscular injection (*p*≤0.05, Figure 2A). In contrast, samples obtained from the spleen of animals with pre-existing immunity that were immunized by the I.N. route contained the highest number of activated IFN-γ secreting cells (815±190 spot-forming cells (SFC)/million mononuclear cells (MNCs), Figure 2C) although this was approximately 30% lower than that seen in naive animals (1,340±146 SFC/million MNCs, Figure 2A). Pre-existing immunity also significantly reduced the number of these cells produced by animals immunized by the I.M. (210±68 SFC/million MNCs) and P.O. routes (71±12 SFC/million MNCs) with respect to those seen in naïve animals (2,145±12 (I.M.) and 885±168 (P.O.)), *p*≤0.05, Figure 2A). A very limited number of activated IFN-γ secreting mononuclear cells were detected in splenocytes of naïve animals immunized with the AdlacZ vector (50±20 SFC/million MNCs, Figure 2A) and those with pre-existing immunity to the same virus (12±1, AdlacZ, Figure 2C). Significant levels of INF-γ positive cells were detected in bronchioalveolar lavage fluid (BAL) after intranasal immunization (146±14 SFC/million MNCs, *p*≤0.05, Figure 2B). This was not significantly compromised by pre-existing immunity to adenovirus (120±16 SFC/million MNCs, *p*≤0.06, Figure 2D). A limited number of INF-γ secreting cells were detected in BAL from naïve animals immunized by the I.M. or P.O. routes (4±2 and 11±5 SFC/million MNCs respectively, Figure 2B) and were similar to that seen in samples obtained from animals given the AdlacZ vector (12±1, negative control). Pre-existing immunity to adenovirus reduced these cell populations further to values that were not statistically significant (1±1 (I.M.), 5±2 (P.O.) and 2±1 (AdlacZ) SFC/million MNCs (*p* = 0.08, Figure 2D)). INF-γ positive cells were found in mesenteric lymph nodes (MLN) and Peyer's Patches (PP) (146±11 and 29±5 SFC/million MNCs, respectively) only after oral vaccination with Ad-ZGP (Figure 2B). Pre-existing immunity, however, reduced production of these cells to 18±12 SFC/million MNCs (MLN) and 16±2 SFC/million MNCs (PP) (Figure 2D).

### Effect of Mucosal Immunization on Localized and Systemic Antibody Production Against Ebola Glycoprotein

**Figure 3:**
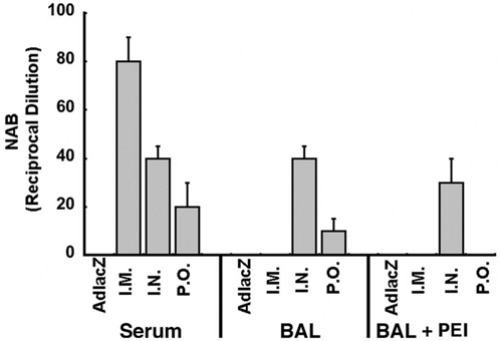
Intranasal Immunization Induces Production of Neutralizing Antibodies in the Lung in the Presence of Pre-Existing Immunity. Forty-five days after immunization, bronchioalveloar fluid was analyzed for the presence of neutralizing antibody by measuring the reduction in infectious titer of ZEBOV-EGFP. The reciprocal dilution plotted for each treatment group (n = 10) reflects the dilution at which the ability of the ZEBOV-EGFP vector to infect target cells was reduced by >50%. Data represent average values obtained from each treatment group and error bars represent the standard deviation of the data. NOTE - Data grouped on the left side of the figure under the label “Serum” is reproduced from Figure 1B of this manuscript and is included here for comparison. PEI - pre-existing immunity to adenovirus serotype 5.

The NAB response was also monitored from bronchioalveolar lavage fluid 45 days post-vaccination. NAB to ZEBOV-EGFP was undetectable in samples obtained from control (AdlacZ) or I.M. immunized mice, whereas levels of 40±10 and 10±5 reciprocal dilution were detected in the BAL of nasally and orally vaccinated animals, respectively (Figure 3). Those with pre-existing immunity and immunized by the nasal route had NAB levels of 30±10. NAB was not detected in mice with pre-existing immunity to Ad5 and immunized by either the intramuscular or oral route.

**Figure 4:**
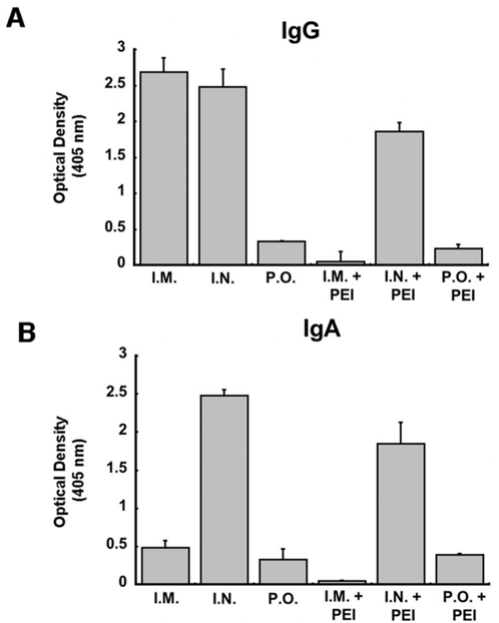
Intranasal Vaccination Induces Anti-Ebola Specific Ig Isotypes in Bronchioalveolar Lavage Fluid in the Absence and Presence of Pre-Existing Immunity. Forty-five days after immunization, bronchioalveolar lavage fluid was analyzed for Ebola-specific IgG (panel A) and IgA (panel B) antibodies by ELISA. The optical densities obtained from each treatment group are presented to serve as a measure of relative concentration. PEI - pre-existing immunity to adenovirus serotype 5.

Further characterization of Ebola glycoprotein-specific immunoglobulin isotypes obtained from bronchioalveolar lavage fluid revealed that pre-existing immunity correlated with a marked decrease in the production of both IgG and IgA antibodies in mice immunized by intramuscular injection (Figure 4A and 4B). IgG and IgA levels were the lowest in that were vaccinated orally regardless of whether they had pre-existing immunity (Figure 4A and 4B). Immunization by the nasal route induced a strong IgG response that was reduced by an average of 25% in the presence of pre-existing immunity. The strongest IgA response was detected in samples obtained from animals given the vaccine by the nasal route. IgA levels, however, were also reduced by 25% in mice with pre-existing immunity to Ad5.

### Effect of Mucosal Immunization on Survival Against Lethal Ebola Challenge in the Presence of Pre-Existing Immunity

**Figure 5:**
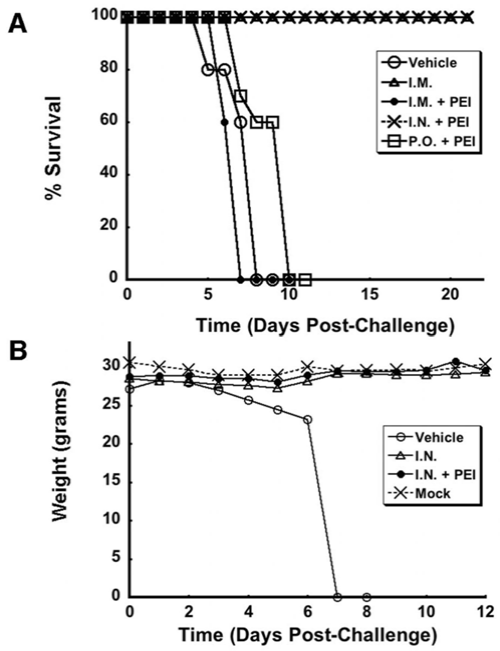
Intranasal Immunization with Recombinant Adenovirus Expressing Ebola Glycoprotein Affords Protection Against Lethal Challenge Even in the Presence of Pre-Existing Immunity. Naïve mice (n = 10) were vaccinated with a single dose of 1×10^10^ particles of adenovirus expressing the Ebola glycoprotein (Ad5-ZGP) by the intramuscular (I.M.), nasal (I.N.) or oral (P.O.) route. Animals in which pre-existing immunity (PEI) was established by I.M. injection of 5×10^10^ particles of adenovirus 5 expressing beta-galactosidase (AdlacZ) were also vaccinated in the same manner. Twenty-eight days later, mice were challenged with 200 LD_50_ of mouse-adapted Ebola virus (Zaire strain). Data represent survival (panel A) and loss of body weight (panel B) over time and is reported as average body weight from each treatment group. Mock - age matched, untreated, unchallenged mice included in this data set to indicate normal weight variation over time. NOTE: Data for naïve mice immunized by the oral route (P.O.) and those with pre-existing immunity and vaccinated by the I.M. route (I.M.+PEI) were not included in this figure for visual clarity. All naive mice immunized orally survived challenge while none in the I.M.+PEI group survived.

The most direct means of evaluating vaccine efficacy in mice is to assess protection by monitoring weight loss and death rates after a lethal challenge of Ebola [43]. Therefore, mice were immunized with 1×10^10^ particles of Ad5-ZGP by the intramuscular (I.M.), intranasal (I.N.) or oral (P.O.) route in the presence or absence of pre-existing immunity. At the time of vaccination, animals had an average anti-adenovirus neutralizing antibody titer of 1∶320 reciprocal dilution. Naïve mice vaccinated by intramuscular injection with saline (vehicle) served as controls for complete lethality following challenge with mouse-adapted Ebola virus. Twenty-eight days after vaccination, animals were challenged with 200 LD_50_ of mouse-adapted (MA)-ZEBOV. Only mice immunized by the nasal route survived the lethal challenge (Figure 5A). This was evident as early as 5 days after challenge when controls and mice immunized by the other routes began to lose weight (Figure 5B). All eventually expired 7 days post-challenge (Figure 5A, some data not shown for clarity). In contrast, naïve mice given the vaccine survived challenge regardless of the route of administration.

### Effect of PEGylation on Improving the Immune Response Achieved by Oral Vaccination

**Figure 6:**
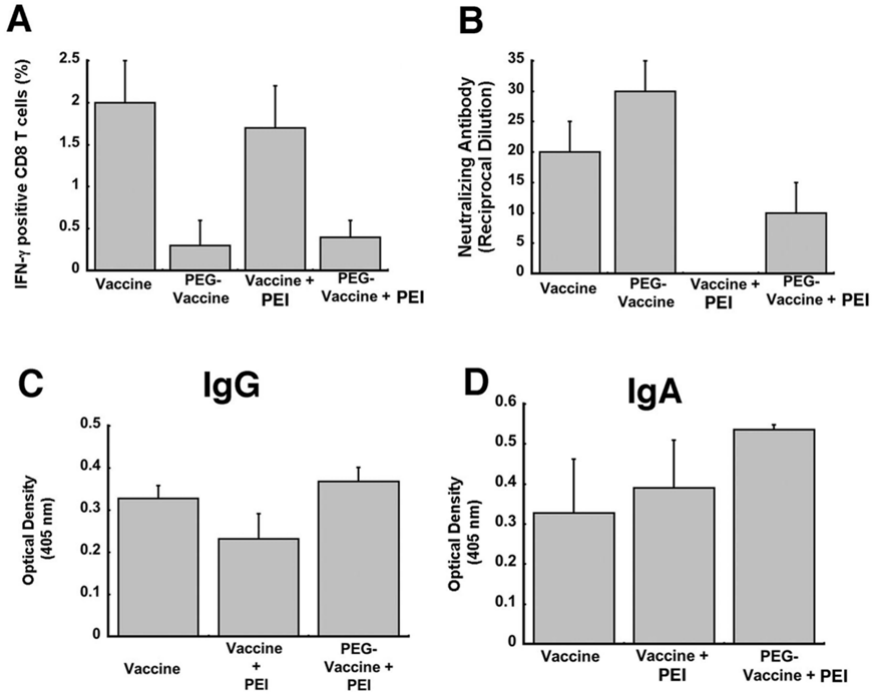
Oral Vaccination with PEGylated Adenovirus Improves the B-cell Mediated but Not the T-cell Mediated Immune Response Against Ebola Glycoprotein. Naïve mice and those with pre-existing immunity were vaccinated with 1×10^10^ particles of either unmodified (Vaccine) or PEGylated (PEG-Vaccine) Ad5-ZGP by oral gavage. Pre-existing immunity (PEI) was induced by I.M. injection with 5×10^10^ particles of adenovirus expressing beta-galactosidase (AdlacZ). (A) Frequency analysis of IFN-γ secreting CD8+ T cells harvested from splenocytes 10 days post-immunization (n = 4/group). (B) Neutralizing antibody (NAB) levels against ZEBOV-EGFP were evaluated 25 days post-vaccination (n = 10/group). (C) Profile of anti-Ebola-specific IgG antibodies. (D) Profile of anti-Ebola-specific IgA antibodies. In all panels, error bars represent the standard deviation of the data.

The studies outlined above strongly suggest that only intranasal immunization can successfully afford protection against Ebola virus in the absence and presence of pre-existing immunity. It was also clear that additional strategies were needed to improve vaccination by either the oral or intramuscular route in the presence of pre-existing immunity. Covalent attachment of activated monomethoxypolyethylene glycol to the protein capsid of the Ad5-ZGP vector was evaluated as a potential way to improve the immune response after oral vaccination. Based upon previous observations, we hypothesized that this modification would protect the virus from neutralization by immune sera and improve survival in gastrointestinal tract [26], [27], [30], [44]. The frequency of Ebola glycoprotein peptide-specific activation of IFN-γ positive CD8+ T cells was not significant in naïve animals given the PEGylated virus (0.3±0.3%) with respect to those given the unmodified virus (2.0±0.5%, *p* = 0.09, Figure 6A). Similar results were also seen in the presence of pre-existing immunity in either treatment group (0.4±0.2% PEGylated vaccine, 1.7±0.5%, unmodified vaccine).

In contrast, NAB levels equivalent to 30±10 reciprocal dilution were detected in naïve animals given the PEGylated vaccine (Figure 6B). Samples obtained from animals given the unmodified virus had levels of 20±5 reciprocal dilution. Animals with pre–existing immunity against adenovirus and treated with the PEGylated preparation were also able to produce detectable levels of NAB (10±5 reciprocal dilution) whereas NAB was not found in sera from mice given the unmodified vector orally. Further characterization of Ebola glycoprotein-specific Ig isotypes revealed that the PEGylated vector could possibly stimulate production of IgG in the presence or absence of pre-existing immunity with respect to levels found in animals given the unmodified vector (Figure 6C). Modification of the virus induced a slight increase of IgA in the presence of pre-existing immunity with respect to that seen in naïve animals given unmodified virus (Vaccine, Figure 6D) and those with pre-existing immunity (Vaccine+PEI). Despite this, animals with pre-existing immunity to adenovirus and immunized with the PEGylated vaccine orally did not survive after challenge with 200 LD_50_ of (MA)-ZEBOV.

## Discussion

With mortality rates as high as 95%, Ebola infection occurs largely by direct contact with blood, tissues or skin of patients, and through mucosal exposure [45], [46]. To date, few efforts have been made to focus on the mucosa as the primary sight of exposure to Ebola virus and priming it for participation in the host defense by vaccination [47]. This is surprising given that the pulmonary, nasal and oral immune systems which comprise the mucosal-associated lymphoid tissues (MALT) are responsible for the production of approximately 80% of all immunocytes [48], [49] and mucosal immunity is often the first line of defense against pathogens coming in contact with susceptible hosts. Many studies in rodents indicate that systemic immunization produces strong anti-viral systemic responses while mucosal vaccination can stimulate both the mucosal and systemic immune systems and can confer long-term immunological memory against a given pathogen despite the fact that the magnitude of these responses are often reported to be somewhat reduced [37], [48], [50]. We have found this to be the case in the studies outlined here since the systemic cellular response and neutralizing antibody levels against Ebola Zaire GP were consistently lower following nasal and oral vaccination. Mucosal vaccination did, however, generate significant cellular and antibody responses in the periphery (BAL, MLN or Peyer's patches) and a single intranasal immunization with Ad5-ZGP conferred 100% protection even in the presence of pre-existing immunity.

Administration of recombinant adenovirus-based vaccines to the mucosa has also conferred sufficient protection against challenge with a variety of pathogens in the presence of pre-existing immunity to the vaccine carrier in mice and other pre-clinical models of disease [5], [19], [32], [51], [52]. This served as a basis for the present study. A single intranasal dose of a recombinant Ad5 vaccine expressing the Zaire Ebola glycoprotein conferred 100% protection in both naïve mice and those with pre-existing immunity despite the fact that the strength of the immune response generated by this route of administration was quantitatively lower than that seen in animals vaccinated by intramuscular injection. It is also important to note that pre-existing immunity induced by intramuscular injection did not severely compromise the T cell-mediated response at either the systemic or mucosal levels in these animals. The level of anti-Ebola GP antibodies in the circulation and in the lung was also not significantly compromised by pre-existing immunity to adenovirus. While these results suggest that intranasal vaccination with an Ad-based vaccine is indeed a promising strategy to overcome pre-existing immunity, one might wonder how accurately our results translate to natural exposure to the wild type virus via the respiratory tract. This is somewhat difficult to establish in the mouse model since the ability of the wild-type adenovirus to replicate is limited [53]. In addition, the amount of neutralizing antibody present in the nasal cavity of those in the general population with pre-existing immunity to adenovirus 5 has not been assessed to the degree that serum neutralizing antibodies have, making it difficult to set a relative parameter for one to target in pre-clinical animal models. Lack of this data may be due to the invasive nature of the technique for acquiring samples and/or the fact that antibody levels in the mucosa of individuals with established pre-existing immunity to adenovirus type 5 are quite low and transient in contrast to systemic levels of anti-adenovirus neutralizing antibodies which are quite robust and persist over time (unreported observations). Thus, for these studies, we decided to induce pre-existing immunity against adenovirus by intramuscular injection at a dose that has been shown to induce production of systemic neutralizing antibodies at 1∶320, a level mostly below what was reported in humans with documented pre-existing immunity [8], [11], [12]. Additional studies designed to assess the amount of neutralizing antibody to AdHu5 in the lung over time after intranasal administration of varying doses of AdHu5 are currently underway in an effort to further define stringent conditions under which pre-existing immunity can be established for experimental testing.

Data obtained from animals vaccinated by the oral route provided several insights about the immunological requirements for protection against Ebola in a mouse model. Immunization by this route induced quantitatively lower T and B cell mediated immune responses against Ebola glycoprotein with respect to that achieved by either intramuscular or intranasal immunization. Despite this, every naïve animal immunized with a single oral dose of Ad-ZGP survived challenge, giving better precision on the minimal threshold of immunity required to achieve protection. As seen with intranasal immunization, the T cell-mediated response was not compromised by pre-existing immunity 10 days after oral vaccination. The number of IFN-γ secreting T cells in both mucosal and systemic compartments of these animals at day 45, however, was significantly reduced by pre-existing immunity. It is possible that the peak T cell response at day 10 does not reflect the extent by which pre-existing immunity decreases the Ad5-ZGP-induced T cell response. Alternatively, it is also possible that the ELISPOT assay could detect subtle variations that flow cytometery could not monitor accurately due to differences in the sensitivity of each assay. Neutralizing antibody was not detected in the serum of animals with pre-existing immunity given a single oral dose of the vaccine. More importantly, none of these animals survived challenge with mouse adapted Ebola.

We attempted to improve the immunogenicity of the adenovirus-based vaccine by protecting it from the harsh environment of the gastrointestinal tract and from neutralization by anti-adenovirus antibodies by PEGylation. Interestingly, the T-cell mediated immune response was significantly reduced and antibody levels increased in naïve animals given a single oral dose of the PEGylated vaccine with respect to that seen in animals given the same dose of unmodified Ad5-ZGP. It has been shown that modification of virus capsids by PEGylation can significantly dampen the T-cell mediated immune response against the virus and stimulate the antibody response against a secreted antigen [26], [27], [30], [54]. Taken together, these data support the notion that the exposition of the virus capsid proteins facilitates the immune response against the encoded antigen. Optimization of PEGylation chemistries and/or densities on adenovirus-based vaccine that promote and strengthen protective immune responses following oral immunization is currently underway.

Delivery of recombinant adenoviral vaccines to either the nasal or intestinal mucosa is an attractive vaccination strategy for many reasons. Vaccines administered in this manner will offer improved safety with respect to disease transmission and needle-stick injuries among health care workers, significant issues of concern in developing countries where the demand for many vaccines is high [55]. Mucosal administration of vaccines reduces the pain associated with vaccination, eliminates the need for specialized training programs for large vaccination campaigns and makes self-administration of the vaccine possible. This route of administration may also significantly reduce systemic toxicity associated with recombinant adenovirus despite the fact that it has been shown that nasal immunization with recombinant adenovirus-based vaccines can facilitate translocation of the virus to the central nervous system [52]. Even though testing of other virus-based vaccines such as influenza have reported similar findings and are currently used in the clinic [56], studies designed to fully assess the toxicological profile of adenovirus vaccine after nasal administration are also currently underway.

We have shown that nasal immunization with an Ad5-based vaccine can induce a long-term protective immune response against Ebola virus in a mouse model which is not impeded by pre-existing immunity to adenovirus serotype 5. While these results are extremely encouraging, further characterization of the immune response against both the encoded antigen and the adenovirus vector in larger, clinically relevant animal models is vital for both understanding the biology of Ad vaccines and for the development of an effective Ebola vaccine suitable to populations with different requirements [57], [58]. The issue of pre-existing immunity must also be adequately addressed in order to develop efficient recombinant adenovirus-based vaccines. While the majority of the literature suggests that pre-existing immunity significantly hamper the effective use of AdHu5 vaccine carriers, other investigators have reported that pre-existing immunity did not interfere with the potency of recombinant Ad5-based vaccines in both pre-clinical models of disease and in humans [59], [60]. Thus, additional studies identifying clinically relevant conditions under which to test Ad-based vaccine candidates are necessary to assess the full impact of pre-existing immunity on vaccine potency, including in different compartments. Better define the role of pre-existing immunity on vaccine-induced immunity will further the understanding of how individuals previously exposed to adenovirus will respond to these immunization regimens.
